# Risk factors, person, place and time characteristics associated with Hepatitis E Virus outbreak in Napak District, Uganda

**DOI:** 10.1186/s12879-017-2542-2

**Published:** 2017-06-26

**Authors:** Geofrey Amanya, Samuel Kizito, Immaculate Nabukenya, Joan Kalyango, Collins Atuheire, Hellen Nansumba, Stephen Akena Abwoye, Denis Nixon Opio, Edrisa Kibuuka, Charles Karamagi

**Affiliations:** 1 Clinical Epidemiology Unit, School of Medicine, P.O Box 7072, Kampala, Uganda; 2 Epidemiology and surveillance Division, Ministry Of Health, P.O Box 7076, Kampala, Uganda; 3 Makerere University College of Health Sciences, P.O Box 7072, Kampala, Uganda

**Keywords:** Hepatitis E, Epidemic, Uganda

## Abstract

**Background:**

Hepatitis E is self-limiting, but can cause death in most at risk groups like pregnant women and those with preexisting acute liver disease. In developing countries it presents as epidemic, in 2014 Hepatitis E Virus (HEV) outbreak was reported in Napak district Uganda. The role of factors in this setting that might have propagated this HEV epidemic, including host, agent, and environmental characteristics, were still not clear. This study was therefore conducted to investigate the risk factors, person, place and time characteristics, associated with the hepatitis E virus (HEV) epidemic in Napak district.

**Methods:**

Review of line lists data for epidemiological description and matched case control study on neighborhood and age in the ratio of 1:2 were used to assess risk factors for HEV outbreak in Napak. Cluster and random sampling were used to obtain a sample size of 332, (111 cases, 221 controls). Possible interaction and confounding was assessed using conditional logistic regression.

**Results:**

Over 1359 cases and 30 deaths were reported during 2013/2014 HEV outbreak. The mean age of patients was 29 ± years, 57.9% of cases were females. Overall case Fatality Ratio was 2.2% in general population but 65.2% in pregnant women. More than 94% of the cases were reported in the sub counties of Napak, 5.7% of cases were reported in the outside neighboring districts. The epidemic peaked in January 2014 and gradually subsided by December 2014. Risk factors found to be associated with HEV included drinking untreated water (OR 6.69, 95% CI 3.15–14.16), eating roadside food (OR 6.11, 95% CI 2.85–13.09), reported not cleaning utensils (OR 3.24, 95% CI 1.55–1.76), and being a hunter (OR 1.14, 95% CI 1.03–12.66).

**Conclusion:**

The results of this study suggest that the virus is transmitted by the feco-oral route through contaminated water. They also suggest that active surveillance and appropriate measures targeting community and routine individual health actions are important to prevent transmission and decrease the deaths.

## Background

Previously in Kitgum, Uganda reported one of the longest recorded outbreaks of Hepatitis E virus (HEV) in the history of the country [1]. HEV outbreaks occur mainly in developing countries where genotypes 1 and 2 are predominant. Although these epidemics are transmitted through feco-oral route, through fecally contaminated drinking water or food, person-to-person transmission has been reported elsewhere [1–3]. In developed countries HEV is anthropozoonotic and cases are mainly sporadic and linked to genotype 3 and 4 [4–6]. Studies of genotype 1 HEV infection have suggested that only 20–30% of infections are symptomatic [7]. Even then, HEV disease is self-limiting and usually does not result in long-term sequaele [5, 8]. The consequences of HEV infections in pregnancy are severe, and have been associated with high case fatality rates (up to 20%). In the general population a Case Fatality Rate (CFR) of 1% and 2% have been proven possible [8–11]. Vaccines have been developed and tested in clinical trials, however these have not been made available for public use [11, 12]. This study aimed at identifying the risk factors, person, place and time characteristics of the 2013/2014 HEV epidemic in Napak district of Uganda.

## Methods

### Study site and subjects

The study was carried out in Napak district, northern Uganda. It has seven sub counties, 31 parishes, and 227 villages. It is predominantly rural-nomadic with an estimated total population of 146,630 persons. Temperatures are varying from 15 to 32.5 °C depending the season. There is no month when rainfall exceeds potential evaporation. The soil are black or dark grey clays with very low organic material, they have a medium moisture storage capacity. In this Karamoja region Napak, deforestation and wild fires have left the vegetation inexistent with no green cover. Three sub counties of Lokopo, Lorengechora and Matany, accounted for the bulk cases, other isolated cases had been identified from the neighboring districts of Moroto, Nakapiripirit, Katakwi and Kotido. From an unpublished Health report in 2014 Napak district had a low latrine coverage of only 18.6% and safe water coverage of 62%. Nevertheless 44% of the safe water sources are non-functional.

### Surveillance data

We obtained line listings data from the office of the district “biostatistician” for all the cases since the beginning of the outbreak from 2013 to 2014. This data was routinely collected for all the patients identified during the outbreak.

### Case control study

We begun conducting a case control study in November 2014 in which we matched the cases with age and geographical location matched controls. A case patient was defined as any individual who was diagnosed with Hepatitis E basing on clinical presentation of jaundice as ascertained by a team of physicians with at least one of the accompanying symptoms (fatigue, anorexia, abdominal pain, arthralgia, fever or headache) and serological evidence of HEV infection [IgM antibody, MP Biomedical Suisse S.A. Switzerland]. The study physician excluded patients If they had jaundice of unknown etiology, or could not be contacted and reported travelled out of Napak during the review period. Case listing data missing variables of interest. Case listing data missing variables of interest, like laboratory results, were also excluded. Potential controls were individuals that had not presented with clinical picture suggestive of hepatitis E since the epidemic began. They were selected from the general population residing in the three sub counties and with each control matched to a case by neighborhood and age (±5 year’s difference) on ratio of 1:2. We used random and cluster sampling to obtain a sample size of 332, (111 cases, 221 controls), 80% power to detect a minimum OR of 2.5, alpha error of 5%, design effect of 2 and a 10% none response rate.

### Data collection and analysis

We developed, translated and pretested a questionnaire into the local language (Karimojong) for easy comprehension of participant’s views and pretested it. We collected information on social demographic information: age, sex, pregnancy status, social economic, location of primary residence, household size (divided into tertiles) and occupation. Other culture-specific practices such as drinking locally made fermented alcoholic brew, attending ceremonies where communal hand washing was common, washing utensils, hand washing practices especially after eating and usage of toilet, the presence as well as use of soap, exposure to various animals including ownership and staying with animals in the same household.

We obtained additional environment information including the primary source of water, availability and treatment of drinking water, the type and number of drinking water storage vessels, latrine availability and use, season (dry or wet) and common bathing practices (common basin with others). Safety and confidentiality of data were ensured through use of unique identification. Collected data was kept under lock and key research assistants were trained. In a household where there were more than two, a tossup was done to select who to be interviewed.

### Statistical analysis

We coded and registered the data into a computer database created using Epi-info version 7.5. We checked for errors, cleaned the data periodically and froze the data. A copy of the data was transfered to STATA version 12 for analysis. Proportions (percentages), a spot map and epidemic curve were used to describe respectively the person, place and time characteristics of the HEV outbreak in Napak district 2013/2014.

To identify the risk factors associated with the HEV outbreak, we carried out conditional logistic regression reporting odds ratios. We performed bivariate analysis and all the variables, with *p* < 0.20 were subjected to multivariate analysis; possible interactions and confounding were assessed.

## Results

### Description of the person, place, and time characteristics of HEV outbreak

**Fig. 1:**
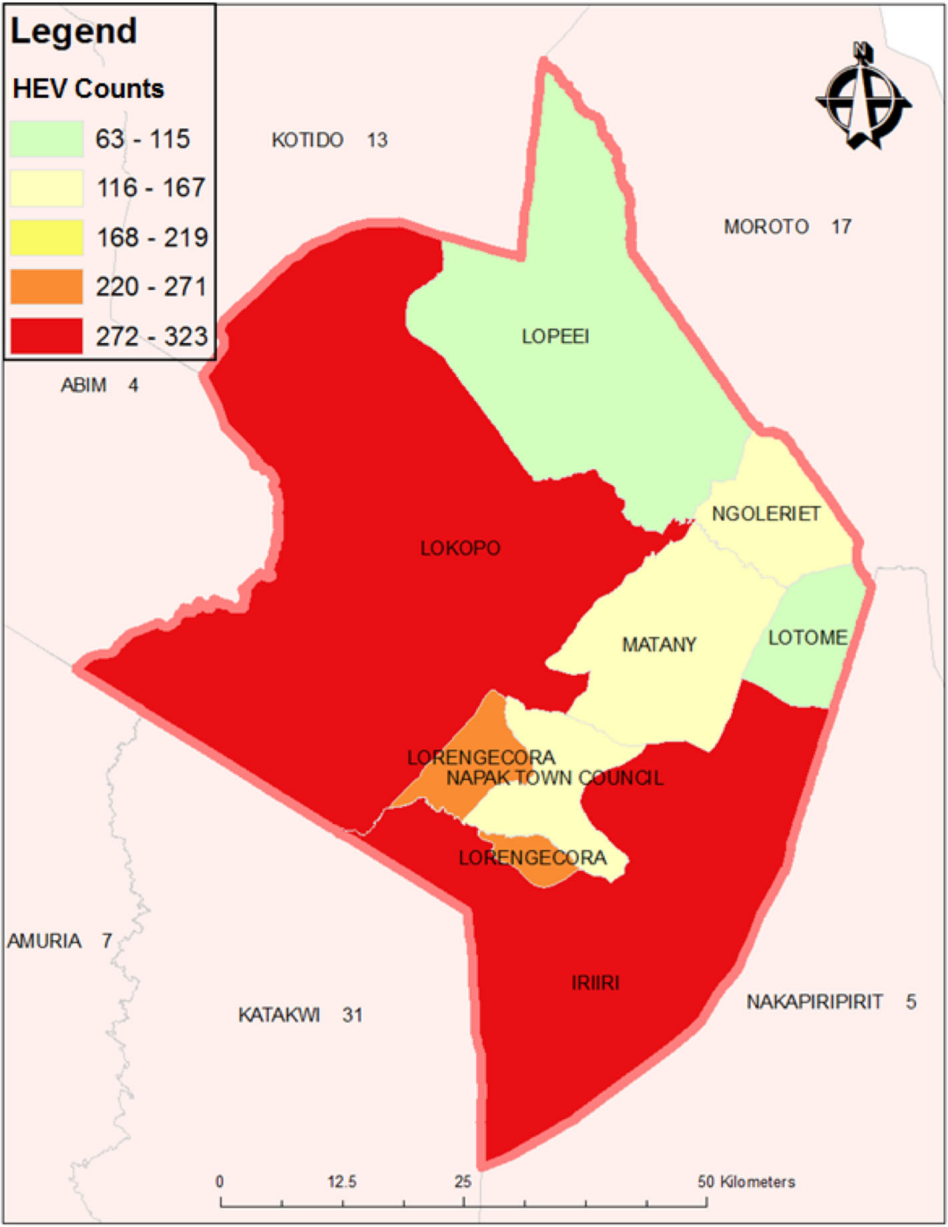
Distribution of confirmed HEV cases in Napak district per Sub County, 2013/2014 *-Spot Map was GIS plotted*

We identified a total of 1359 cases of HEV infection from Napak district listings during this epidemic (Fig. 1). The mean age of patients was 29 ± years and 57.9% (788) of the HEV cases were females. The overall CFR was 2.2%, it was highest in the 18–30 years age group (3.9%) and females (3.3%). The CFR was highest in pregnant women at 65.2%. Details are highlighted in Table 1.

**Table 1:** Description of the person characteristics of HEV outbreak 2013/2014

Characteristic		Proportion of Cases *n* (%)	Died	CFR (%)
Age in years	<=5	51 (3.8)	0	0
	6–17	180 (13.3)	2	1.1
	18–30	594 (43.7)	23	3.9
	31–59	445 (32.7)	4	1
	60>=	89 (6.5)	0	0
Sex	Female	788 (58.0)	26	3.3
	Male	571 (42.0)	4	0.7
Pregnancy Status^a^	Yes	23 (1.7)	15	65.2
Overall Total		1359	30	2.2

^a^Subset of females who were pregnant

**Fig. 2:**
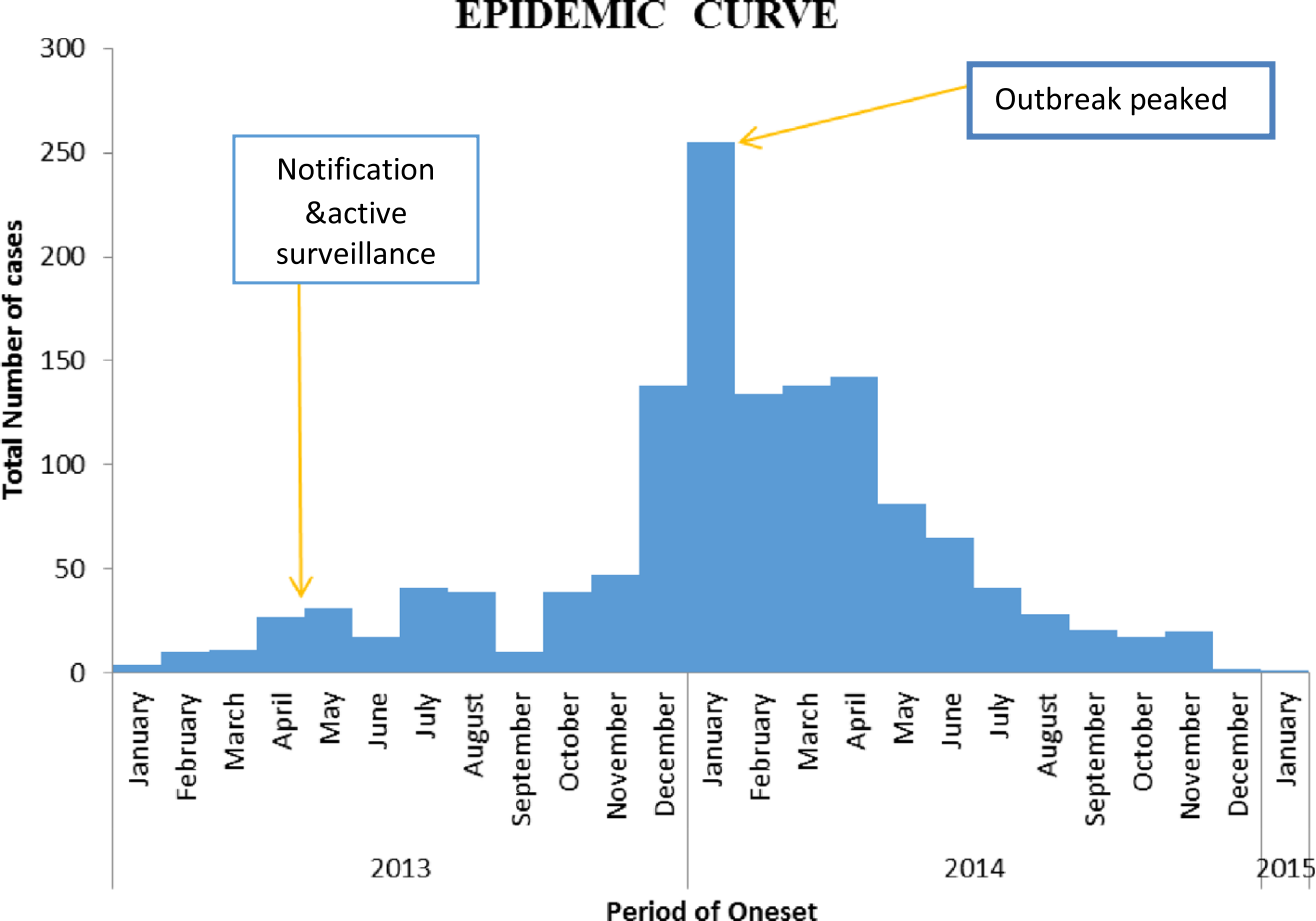
Distribution of confirmed HEV cases by month of onset, Napak district (2013/2014)

Cases of HEV were first reported in January 2013 while the notification and surveillance for the epidemic began in April 2013. The HEV epidemic peaked in January 2014 and gradually subsided. By December 2014, there were no more cases reported. The median incubation period was 40 days IQR (24–53) as shown in Fig. 2.

In Table 2 **,** Lorengechora and Ngolereit had the highest proportion of the cases in other sub counties like Lopeei (0.8%), Iriri (0.8%), Matany (0.7%), Lotome (0.5%) and Lokopo (0.02%) the CFR were below 1% of the total population.

**Table 2:** Proportion of HEV cases per sub county in Napak during 2013/2014 outbreak

Sub counties	Population estimates^a^	Cases	Proportion of cases %
Lorengchora	11,099	233	2.01
Ngolereit	10,502	126	1.20
Lopeei	13,393	104	0.77
Iriri	41,932	323	0.77
Matany	22,810	159	0.70
Lotome	11,589	63	0.54
Lokopo	21,311	288	0.02

^a^Census 2014 UNHPS

### Case control study

Four cases and 7 controls were excluded because we could not contact them. We analyzed data from a total of 107 cases, and 214 controls. With the exception of sex, the cases and controls were comparable at baseline. At bivariate (simple) analysis HEV was associated with drinking untreated water (OR 5.19, 95% CI 2.82–9.54), not cleaning their utensils (OR 2.72, 95% CI 1.60–4.61), unsanitary (open) defecation practices (OR 2.46, 95% CI 1.47–4.12), reported frequent travels OR 2.04, 95% CI (1.14–3.63), “never” washed hands with soap after visiting the latrine (OR 1.64, 95% CI 1.01–2.65), never using soap to wash their hands before eating (OR 1.46, 95% CI 1.47–4.12), those with a well (OR 10.7, 95% CI (1.15–98.5). As shown in Table 3.

**Table 3:** Bivariate analysis of independent factors associated with Hepatitis E Virus in Napak, 2013/2014

Variable	Cases	Controls	Odds Ratio	*P*-value
	*N* = 107(%)	*N* = 214(%)	(95%CI)	
Male Sex	37 (34.6)	104 (48.6)	0.54 (0.3–0.87)	0.017^*^
Female not pregnant	19 (27.1)	33 (30.0)	1.55 (0.65–3.43)	0.337
Dry season	68 (63.5)	150 (70.1)	1.49 (0.83–2.52)	0.188
Live in rural setting	94 (87.8)	194 (90.6)	1.00 (0.28–3.50)	1.000
Education level				
None	78 (72.9)	172 (80.4)	1.0	
Primary	19 (17.7)	30 (14.0)	1.46 (0.75–2.85)	0.263
Secondary	7 (6.5)	9 (4.2)	2.32 (0.65–8.29)	0.193
Tertiary	3 (2.8)	3 (1.4)	2.53 (0.49–3.14)	0.267
Unsanitary (Open defecation) habits	77 (71.9)	111 (51.9)	2.46 (1.47–4.12)	0.001^*^
Drunk alcohol 2 weeks before outbreak.	80 (74.7)	151 (70.5)	1.24 (0.73–2.21)	0.420
No soap in the household.	56 (52.3)	89 (42.6)	1.59 (0.98–2.59)	0.060
Never wash hands with soap after defecation.	50 (46.7)	70 (35.0)	1.64 (1.01–2.65)	0.043^*^
Clean hands with soap before eating	69 (64.5)	141 (65.9)	0.93 (0.55–1.57)	0.789
Did not clean utensils after eating food.	70 (65.4)	94 (44.9)	2.72 (1.60–4.61)	*P* < 0.001^*^
Drinking untreated water.	58 (54.2)	181 (84.5)	5.19 (2.82–9.54)	*P* < 0.001^*^
Had contact with animals prior to epidemic.	61 (57.0)	132 (61.7)	0.81 (0.49–1.32)	0.402
Attended funerals 2 weeks before outbreak.	13 (16.6)	14 (9.2)	0.56 (0.55–3.02)	0.556
use the same basin for washing hands	42 (39.3)	75 (35.1)	1.24 (0.73–2.10)	0.420
Ate food from the roadside	43 (40.2)	39 (18.2)	2.88 (1.70–4.84)	*P* < 0.001^*^
Primary source of water				
Tap water	19 (17.8)	49 (22.9)	1.0	
Bore Hole	82 (76.6)	162 (75.7)	1.34 (0.69–2.58)	0.381
Well	5 (4.7)	2 (1.0)	10.7 (1.15–98.5)	0.037^*^
Dam	1 (1.0)	1(.5)	2.16 (0.13–34.9)	0.586
Household has no latrine	82 (76.6)	173 (80.4)	0.76 (0.42–1.37)	0.355
Have common bathing practices	59 (58.1)	124 (57.9)	1.16 (0.68–1.99)	0.587
Had recently travelled	27 (25.20)	30 (14.0)	2.04 (1.14–3.63)	0.016^*^
Did you own or have contacts with animals prior to the epidemic				
No	46 (43.0)	82 (38.3)		
Yes	61 (57.0)	132 (61.7)	0.81 (0.49–1.32)	0.402
If yes which animals				
Pigs	3 (4.9)	9 (6.7)	1	
Cows	24 (39.3)	38 (28.4)	1.59 (0.32–7.88)	0.570
Goats	15 (24.6)	39 (29.1)	1.36 (0.24–7.67)	0.720
Poultry	19 (31.1)	48 (35.8)	0.82 (0.18–3.70)	0.790

^*^Significant *P* < 0.05.Univariate conditional logistics comparing cases and controls

In Napak, livestock ownership is common. In this study livestock ownership was 57% among cases and 61.7% among controls. Although contact or ownership with animals were reported, there was no significant association between HEV and ownership of animals or contact with them (Table 3). We also explored for possible interactions since there was no prior knowledge of possible interaction between predictor variables. Thus, all predictor variables were Included in the model, However, there was No signifi­cant interactions were seen in relation to HEV between Sex, unsanitary defection practices, Presence of soap in Household, Cleaning utensils and hands after eating, untreated water at *P* < 0.05.

Variables like education were confounding washing of utensils and primary source of water and season were confounding occupation. At multivariate conditional logistic regression factors that were independently associated with HEV included drinking untreated water (OR 6.69, 95% CI 3.15–14.16), eating roadside food (OR 6.11, 95% CI 2.85–13.09), not cleaning utensils (OR 3.24, 95% CI 1.55–3.76) and being a hunter (OR 1.14, 95% CI 1.03–12.66). Details are shown in Table 4.

**Table 4:** Multivariate analysis of independent factors associated with Hepatitis E Virus, Napak (2013/2014)

Variable	Odds Ratio	95% CI	*P*-Value
In the week before the epidemic did you eat roadside food(Yes)	6.11	2.85–13.09	*P* < 0.001*
Did you drink any untreated water(Yes)	6.69	3.15–14.16	*P* < 0.001*
Do you always clean your utensils(No)	3.24	1.55–3.760	0.002*
Education Level			
Primary	1.57	0.67–3.64	0.293
Secondary	5.57	0.94–33.0	0.059
Tertiary	1.54	0.24–9.98	0.650
Primary source of water			
Borehole	0.93	0.40–2.14	0.865
Well	2.86	0.25–32.2	0.394
Dam	1.76	0.09–31.9	0.702
Availability of soap in household(No)	1.78	0.88–3.59	0.106
Season(Wet)	1.42	0.67–3.02	0.368
Occupation-Hunter	1.14	1.03–12.66	0.013*

NB: The model was determined by means of conditional logistic regression, all variables adjusted for age, OR, confidence interval and *P*-Values

## Discussion

### Descriptive epidemiology

The burden of HEV outbreak is reflected by proportional morbidity from HEV at facilities and in communities. A median number of 34 cases were reported daily, female patients were observed to be more likely to be affected by the HEV outbreak. A study in Northern Namibia indicates that among patients with HEV male patients predominated over female cases 2.5:1 [13]. This distribution in females might be attributed to more severe clinical presentation in women, especially those who are pregnant [3, 8, 14]. High proportions of cases (43.7%) were adults aged between 18 and 30 years, this may be due to the culture differences where adults mainly search for livelihoods [15]. Contrary to these findings, in a study carried out in North India [16] showed 3.8% of the cases were aged below 5 years, indicating that also under-fives are at risk of HEV.

The high case fatality rate in the study (2.2%) is higher than that reported earlier in Uganda in 2008 (1.5%) [17], but lower than that reported in Darfur in 2004 (1.7%) [18]. With proper case management a lower CFR (<1%) could have been achieved [1]. This is likely to be due to frequent migration between dry seasons, in search for settlement areas. In this study, extremely high CFR of 65.2% was reported among pregnant women. This is higher than the 48% reported in sporadic cases in Ethiopia [6] and the 12.5% reported in Kenya in 2012 [19]. Differences in environmental factors and lack of community awareness on harmful traditional medicine like the use of local herbs to treat some complications during pregnancy could have contributed high CFR. Lorengechora being a town council, cases were most likely to be transmitted by eating roadside food [1, 2, 9]. With unsanitary food handling methods and exposure to many people, risk for HEV would be high. Ngolereit is in the north, neighboring Moroto, a very highly nomadic setting, movement could have enhanced transmission of HEV virus evidenced by number of cases reported out.

The epidemic curve was multimodal and suggestive of a propagated point source common vehicle epidemic, with the largest peak registered in the 48th epidemiologic week. Temporal peaks occurred during the 43-48th epidemiological week, coinciding with the rainy season and flooding in January. Flooding could have carried away any potential fecal substances causing contamination. Studies done in Ethiopia 2012 [6], and Uganda [2, 3] showed high proportions of cases occur in a dry season.

### Risk factors for HEV outbreak

At multivariate analysis, HEV was significantly associated with eating roadside food, drinking untreated water, failure to wash utensils and being a hunter (Table 4). All these factors suggest that in this study, HEV was transmitted by the feco-oral mechanism through a waterborne route, which is typical of genotype 1 and genotype 2 as previously reported by different studies [2, 7].

The odds of having hepatitis E increased by six times among those eating roadside food. Roadside foods are easily contaminated because of unsanitary food handling methods and exposure to many people and winds. Study carried in Europe [5], confirmed food borne contamination in food handler working in a restaurant with high at risk foods.

Drinking untreated water increased the risk of HEV by almost seven times amongst the cases. This suggests that there was prior contamination of water sources and possible water-borne transmission in Napak district. This is not surprising since the situation regarding water and sanitation in this setting is very poor, this finding is confirmed elsewhere [20–22]. However, no associations with poor water and sanitation situation are reported in sporadic cases Turkmenistan [6]. Not cleaning utensils increased the risk of HEV by more than three times among the cases, unclean utensils could have acted as a reservoir for HEV. This situation was attributed to distant water sources and the scarcity of water in the areas. Cases were more likely to travel outside their homes although this finding was not significantly associated with HEV perhaps due to small numbers in strata. Previous studies have linked HEV to travel [19, 20].

Frequent travelers revealed a history of visiting an endemic country with high-risk enteric exposures prior to infection. In this study, the HEV epidemic is attributed to the nomadic livelihoods of the people in search for water, and life in camps during the dry seasons [2, 8, 19].

Unlike previous reports [1], having more than five members (larger family size) in the household was not significantly associated with HEV. These findings concur with the previous HEV outbreak in Uganda, which did not find any significant association with household size [3]. The findings in recent outbreaks in Uganda and studies done in rural Bangladesh [7, 23] and in India [14] demonstrated a strong role of person-to- person transmission in sustaining the epidemic [16]. In this study it was not evident but may have occurred. Use of unsanitary latrines i.e. like open defecation was associated with HEV at bivariate but not at multivariate analysis, perhaps due to small numbers in the strata. However previous studies from Nepal-India and Uganda confirmed a strong association between use of unsanitary latrines and HEV most probably due to fecal matter being easily washed to water sources [17, 24].

HEV was associated amongst those who reported not washing hands with soap after defecation, whose primary source of water was a well, Promiscuous defecation and improper disposal of children’s feaces could have contributed to the situation. Attending social events was significantly associated with HEV at bivariate but not at multivariate analysis.

Social events like birth ceremonies, funeral ceremonies, visiting shrines and marriages can cause transmission of disease particularly through contaminated food and water [2, 11]. About 40 cases of jaundice were reported among people who attended a traditional function in Lorikitae village, in Matany Sub County. In this study, livestock ownership was high and contact with animals was common. Participants reported owning pigs (6.5%), cattle (31.8%), goats (27.9%) and chicken (34.3%).

Use of Cow dung’ is common, this practice consists of drying the cow dung into pieces which are then heated then used as a preservative for Beans and ataapa (Millet), what remains used for cooking. The same cow dung while still wet is used for construction of houses (Manyatas), these are considered potentially hazardous.

However, there was no significant association between HEV and livestock ownership or contact with animals at multivariate analysis. This contrasts the study done in Ghana, which reported 37% sero-prevalence in pig handlers, suggesting that the predominant HEV genotype were zoonotic [7, 25].

The less educated were more likely to confound not washing utensils. They reported poor hygiene practices, unsanitary disposal of fecal matter and also they never had permanent sources of water. Contamination was possible in these groups during the wet seasons. Hunters who had reported frequent movents were at a higher risk of HEV compared to their to their counterparts who had no occupation.

## Limitations

This data had at least three limitations. First, the data was reported several months after the outbreak so they are subject to possible recall bias which might affect individual’s response. Nevertheless a reference period prior the outbreak was to minimize the bias. Second, in this study we did not take water samples for detection of HEV RNA. However we were able to demonstrate and arrive at the associations in this study using the risk factors. Possible Misclassification Bias since there were no serological test done for controls, but research assistants had a clinical background, thorough screening was done to minimize potential for this bias. Third these investigations might have been susceptible to responder bias because of recently introduced interventions. This could have led to underestimation of our associations.

## Conclusions

Findings from this study suggest the existence of a high proportion of cases of HEV infection in the communities of Napak district in Uganda. The detection of HEV was highly suggestive of circulation of cases in this rural population and to the neighboring districts. This epidemic curve demonstrated propagated multi model suggestive of an indirect transmission with higher proportion of cases reported in female and in adults. A high CFR was reported in pregnant women.

Results from the case-control study demonstrate that eating roadside food, drinking untreated water, not cleaning utensils and hunting was associated with increased risk of HEV infection. This suggests transmission by feco-oral route through contaminated water. Surveillance of HEV through mandatory reporting of communicable diseases should continue to enable detection and timely investigation of emerging and reemerging infections. This study supports the need of interventions like vaccines for the most at risk vulnerable groups and other interventions targeting groups such as community health education talks, and routine individual health on treating drinking water are for prevention of HEV transmission.

## Abbreviations

CEU: Clinical Epidemiology Unit
CFR: Case fertility rate
CI: Confidence interval
DHO: District health officer
HEV: Hepatitis E Virus
IQR: Inter-quartile range
MoH: Ministry of Health
OR: Odds ratio
RNA: Ribonucleic acid
SOMREC: School of Medicine Research Ethics Committee
UNCST: Uganda National Council for Science and Technology
VHT: Village health Team
